# Supplementary material for: Calcium salts of long-chain fatty acids from linseed oil decrease methane production by altering the rumen microbiome in vitro
Source: PLoS One. 2020 Nov 10;15(11):e0242158. doi: 10.1371/journal.pone.0242158 (PMC7654805; doi:10.1371/journal.pone.0242158)
Supplement: S1 Table — 1 CON, non-supplementation; FAL, 2.25%DM calcium salt of long-chain fatty acid supplementation; FAH, 4.50%DM calcium salt of long-chain fatty acid supplementation; FUM, fumarate supplementation; MON, monensin supplementation. 2 Phylum and genus exhibited significant differences (adjusted P < 0.05) identified using DESeq2 with ≥ 1% relative abundance in more than one treatment. 3 The p-value was adjusted using the Benjamini-Hochberg procedure. (DOCX) [file pone.0242158.s001.docx]

**S1 Table Differential abundance in specific taxa at phylum and genus level.**

| Group compared^1^ | Phylum^2^ | Genus^2^ | Log2 Fold change | Adjusted P-value^3^ |
| --- | --- | --- | --- | --- |
| CON vs FAL | Actinobacteria |  | -1.610 | 0.00580 |
|  | Bacteroidetes |  | -0.291 | 0.00074 |
|  |  | *Bacteroidales* BS11 gut group | 2.110 | 0.00222 |
|  |  | *Muribaculaceae* uncultured rumen bacterium | -1.969 | 0.02275 |
|  |  | *Prevotella.1* | -0.756 | <0.00001 |
|  |  | *Prevotellaceae* Ga6A1 group | -1.855 | 0.00001 |
|  |  | *Prevotellaceae* unclassified | -0.565 | 0.03347 |
|  |  | *Rikenellaceae* RC9 gut group | 1.203 | <0.00001 |
|  |  | *Bacteroidales* unclassified | 3.628 | <0.00001 |
|  | Firmicutes | *Streptococcus* | -4.042 | <0.00001 |
|  |  | *Ruminococcus.2* | 0.896 | 0.00432 |
|  |  | *Megasphaera* | -2.062 | 0.00004 |
|  |  | *Selenomonas.1* | -0.661 | 0.03539 |
|  | Proteobacteria |  | -0.910 | 0.00004 |
|  |  | *Succinivibrio* | -1.403 | <0.00001 |
| CON vs FAH | Euryarchaeota |  | 1.311 | 0.00122 |
|  |  | *Methanobrevibacter* | 1.748 | 0.00001 |
|  | Actinobacteria |  | -2.756 | <0.00001 |
|  | Bacteroidetes | *Bacteroidales* BS11 gut group | 4.607 | <0.00001 |
|  |  | F082 uncultured bacterium | 8.215 | <0.00001 |
|  |  | F082 uncultured.rumen.bacterium | 6.902 | <0.00001 |
|  |  | *Muribaculaceae* uncultured rumen bacterium | -2.805 | 0.00015 |
|  |  | *Prevotella.1* | -0.446 | 0.00336 |
|  |  | *Prevotellaceae* Ga6A1 group | -2.525 | <0.00001 |
|  |  | *Rikenellaceae* RC9 gut group | 2.988 | <0.00001 |
|  |  | *Bacteroidales* unclassified | 6.594 | <0.00001 |
|  | Firmicutes |  | -0.796 | 0.00019 |
|  |  | *Streptococcus* | -7.762 | <0.00001 |
|  |  | *Lachnospiraceae NK3A20 group* | 1.197 | 0.00075 |
|  |  | *Ruminococcus.2* | 8.335 | <0.00001 |
|  |  | *Succiniclasticum* | -1.497 | 0.00001 |
|  |  | *Megasphaera* | -3.679 | <0.00001 |
|  |  | *Selenomonas.1* | -1.268 | <0.00001 |
|  | Proteobacteria |  | -2.097 | <0.00001 |
|  |  | *Ruminobacter* | -1.197 | 0.00001 |
|  |  | *Succinivibrio* | -2.392 | <0.00001 |
|  | Synergistetes |  | -0.739 | 0.00917 |
| CON vs FUM | Bacteroidetes | *Prevotella.1* | 0.458 | 0.01494 |
|  |  | *Bacteroidales* unclassified | 1.768 | 0.02361 |
|  | Firmicutes |  | -0.603 | 0.03625 |
|  |  | *Ruminococcus.2* | 0.773 | 0.02608 |
|  |  | *Succiniclasticum* | 2.308 | <0.00001 |
|  |  | *Schwartzia* | -1.685 | <0.00001 |
|  |  | *Selenomonas.1* | -0.772 | 0.01775 |
|  | Proteobacteria | *Ruminobacter* | 1.104 | 0.00034 |
|  |  | *Succinivibrio* | -1.004 | 0.00052 |
| CON vs MON | Actinobacteria |  | -1.831 | 0.00049 |
|  | Bacteroidetes |  | 0.449 | <0.00001 |
|  |  | *Bacteroidales* BS11 gut group | 5.461 | <0.00001 |
|  |  | F082 uncultured.rumen.bacterium | 9.596 | <0.00001 |
|  |  | *Muribaculaceae* uncultured rumen bacterium | -4.028 | <0.00001 |
|  |  | *Prevotella.1* | 0.481 | 0.00147 |
|  |  | *Prevotellaceae* YAB2003 group | 2.296 | <0.00001 |
|  |  | *Rikenellaceae* RC9 gut group | 4.225 | <0.00001 |
|  |  | *Bacteroidales* unclassified | 9.144 | <0.00001 |
|  | Firmicutes |  | -0.843 | 0.00004 |
|  |  | *Lachnospiraceae NK3A20 group* | 2.231 | <0.00001 |
|  |  | *Ruminococcus.2* | 7.923 | <0.00001 |
|  |  | *Succiniclasticum* | -1.566 | <0.00001 |
|  |  | *Anaerovibrio* | -1.291 | <0.00001 |
|  |  | *Megasphaera* | -3.935 | <0.00001 |
|  |  | *Schwartzia* | -0.909 | 0.00189 |
|  |  | *Selenomonas.1* | -2.248 | <0.00001 |
|  |  | *Veillonellaceae* UCG.001 | -1.602 | 0.00289 |
|  |  | *Veillonellaceae* uncultured | -1.262 | 0.00243 |
|  |  | *Veillonellaceae* unclassified | -1.098 | 0.00015 |
|  | Proteobacteria |  | -0.780 | 0.00022 |
|  |  | *Ruminobacter* | -0.671 | 0.01854 |
|  |  | *Succinivibrio* | -0.747 | 0.00459 |
|  | Synergistetes |  | -1.220 | <0.00001 |
|  |  | *Pyramidobacter* | -1.196 | <0.00001 |
| FAL vs FAH | Actinobacteria |  | -1.146 | 0.04341 |
|  | Bacteroidetes |  | 0.199 | 0.02265 |
|  |  | *Bacteroidales* BS11 gut group | 2.497 | 0.00336 |
|  |  | F082 uncultured bacterium | 8.012 | <0.00001 |
|  |  | F082 uncultured.rumen.bacterium | 6.974 | <0.00001 |
|  |  | *Rikenellaceae* RC9 gut group | 1.784 | <0.00001 |
|  |  | *Bacteroidales* unclassified | 2.967 | 0.00038 |
|  | Firmicutes |  | -0.843 | 0.00004 |
|  |  | *Streptococcus* | -3.719 | <0.00001 |
|  |  | *Ruminococcus.2* | 7.438 | <0.00001 |
|  |  | *Succiniclasticum* | -1.516 | 0.00001 |
|  |  | *Anaerovibrio* | -0.726 | 0.00803 |
|  |  | *Megasphaera* | -1.617 | 0.00077 |
|  | Proteobacteria |  | -0.780 | 0.00022 |
|  |  | *Ruminobacter* | -0.969 | 0.00094 |
|  |  | *Succinivibrio* | -0.989 | 0.00028 |
|  | Synergistetes |  | -1.220 | <0.00001 |
|  |  | *Pyramidobacter* | -0.960 | 0.00024 |
| FAL vs FUM | Bacteroidetes |  | 0.221 | 0.01699 |
|  |  | *Bacteroidales* BS11 gut group | -3.426 | <0.00001 |
|  |  | F082 uncultured bacterium | -1.673 | 0.04029 |
|  |  | *Prevotella.1* | 1.214 | <0.00001 |
|  |  | *Prevotellaceae* Ga6A1 group | 2.595 | <0.00001 |
|  |  | *Rikenellaceae* RC9 gut group | -0.883 | 0.00109 |
|  |  | *Bacteroidales* unclassified | -1.859 | 0.01889 |
|  | Firmicutes |  | -0.595 | 0.01443 |
|  |  | *Streptococcus* | 3.509 | <0.00001 |
|  |  | *Succiniclasticum* | 2.288 | <0.00001 |
|  |  | *Megasphaera* | 2.194 | 0.00002 |
|  |  | *Schwartzia* | -1.604 | <0.00001 |
|  |  | *Veillonellaceae* unclassified | -0.897 | 0.00930 |
|  | Proteobacteria |  | 0.607 | 0.01443 |
|  |  | *Ruminobacter* | 1.331 | <0.00001 |
| FAL vs MON | Bacteroidetes |  | 0.741 | <0.00001 |
|  |  | *Bacteroidales* BS11 gut group | 3.350 | 0.00003 |
|  |  | F082 uncultured.rumen.bacterium | 9.667 | <0.00001 |
|  |  | *Muribaculaceae* uncultured rumen bacterium | -2.060 | 0.00665 |
|  |  | *Prevotella.1* | 1.237 | <0.00001 |
|  |  | *Prevotellaceae* Ga6A1 group | 1.861 | <0.00001 |
|  |  | *Prevotellaceae* YAB2003 group | 2.747 | <0.00001 |
|  |  | *Rikenellaceae* RC9 gut group | 3.022 | <0.00001 |
|  |  | *Bacteroidales* unclassified | 5.517 | <0.00001 |
|  | Firmicutes |  | -0.835 | 0.00006 |
|  |  | *Streptococcus* | 4.913 | <0.00001 |
|  |  | *Lachnospiraceae NK3A20 group* | 1.538 | 0.00003 |
|  |  | *Ruminococcus.2* | 7.027 | <0.00001 |
|  |  | *Succiniclasticum* | -1.586 | <0.00001 |
|  |  | *Anaerovibrio* | -1.807 | <0.00001 |
|  |  | *Megasphaera* | -1.873 | 0.00003 |
|  |  | *Schwartzia* | -0.829 | 0.00619 |
|  |  | *Selenomonas.1* | -1.587 | <0.00001 |
|  |  | *Veillonellaceae* UCG.001 | -2.027 | 0.00017 |
|  |  | *Veillonellaceae* uncultured | -1.587 | 0.00017 |
|  |  | *Veillonellaceae* unclassified | -1.271 | 0.00002 |
|  | Proteobacteria | *Succinivibrio* | 0.656 | 0.01634 |
|  | Synergistetes |  | -1.621 | <0.00001 |
|  |  | *Pyramidobacter* | -1.761 | <0.00001 |
| FAH vs FUM | Euryarchaeota |  | -1.012 | 0.02163 |
|  |  | *Methanobrevibacter* | -1.212 | 0.00324 |
|  | Actinobacteria |  | 1.699 | 0.00223 |
|  | Bacteroidetes | *Bacteroidales* BS11 gut group | -5.922 | <0.00001 |
|  |  | F082 uncultured bacterium | -9.685 | <0.00001 |
|  |  | F082 uncultured.rumen.bacterium | -6.957 | <0.00001 |
|  |  | *Muribaculaceae* uncultured rumen bacterium | 1.985 | 0.00991 |
|  |  | *Prevotella.1* | 0.904 | <0.00001 |
|  |  | *Prevotellaceae* Ga6A1 group | 3.265 | <0.00001 |
|  |  | *Rikenellaceae* RC9 gut group | -2.667 | <0.00001 |
|  |  | *Bacteroidales* unclassified | -4.826 | <0.00001 |
|  | Firmicutes | *Streptococcus* | 7.229 | <0.00001 |
|  |  | *Ruminococcus.2* | -7.562 | <0.00001 |
|  |  | *Succiniclasticum* | 3.805 | <0.00001 |
|  |  | *Megasphaera* | 3.811 | <0.00001 |
|  |  | *Schwartzia* | -1.299 | <0.00001 |
|  |  | *Veillonellaceae* unclassified | -0.630 | 0.04921 |
|  | Proteobacteria |  | 1.794 | <0.00001 |
|  |  | *Ruminobacter* | 2.301 | <0.00001 |
|  |  | *Succinivibrio* | 1.389 | <0.00001 |
|  | Synergistetes |  | 0.929 | 0.00121 |
|  |  | *Pyramidobacter* | 0.762 | 0.00216 |
| FAH vs MON | Euryarchaeota | *Methanobrevibacter* | -1.030 | 0.01606 |
|  | Bacteroidetes |  | 0.542 | <0.00001 |
|  |  | F082 uncultured bacterium | -6.880 | <0.00001 |
|  |  | *Prevotella.1* | 0.928 | <0.00001 |
|  |  | *Prevotellaceae* Ga6A1 group | 2.531 | <0.00001 |
|  |  | *Prevotellaceae* YAB2003 group | 2.199 | <0.00001 |
|  |  | *Rikenellaceae* RC9 gut group | 1.238 | <0.00001 |
|  |  | *Bacteroidales* unclassified | 2.550 | 0.03916 |
|  | Firmicutes | *Streptococcus* | 8.632 | <0.00001 |
|  |  | *Lachnospiraceae NK3A20 group* | 1.034 | 0.00742 |
|  |  | *Anaerovibrio* | -1.080 | <0.00001 |
|  |  | *Selenomonas.1* | -0.981 | 0.00020 |
|  |  | *Veillonellaceae* UCG.001 | -1.735 | 0.00152 |
|  |  | *Veillonellaceae* uncultured | -1.408 | 0.00081 |
|  |  | *Veillonellaceae* unclassified | -1.004 | 0.00079 |
|  | Proteobacteria |  | 1.317 | <0.00001 |
|  |  | *Succinivibrio* | 1.645 | <0.00001 |
|  | Synergistetes | *Pyramidobacter* | -0.801 | 0.00089 |
| FUM vs MON | Bacteroidetes |  | 0.520 | <0.00001 |
|  |  | *Bacteroidales* BS11 gut group | 6.776 | <0.00001 |
|  |  | F082 uncultured bacterium | 2.804 | 0.00002 |
|  |  | F082 uncultured.rumen.bacterium | 9.650 | <0.00001 |
|  |  | *Muribaculaceae* uncultured rumen bacterium | -3.208 | 0.00001 |
|  |  | *Prevotellaceae* YAB2003 group | 2.306 | <0.00001 |
|  |  | *Rikenellaceae* RC9 gut group | 3.905 | <0.00001 |
|  |  | *Bacteroidales* unclassified | 7.376 | <0.00001 |
|  | Firmicutes | *Lachnospiraceae NK3A20 group* | 1.650 | 0.00001 |
|  |  | *Ruminococcus.2* | 7.150 | <0.00001 |
|  |  | *Succiniclasticum* | -3.874 | <0.00001 |
|  |  | *Anaerovibrio* | -1.326 | <0.00001 |
|  |  | *Megasphaera* | -4.066 | <0.00001 |
|  |  | *Schwartzia* | 0.775 | 0.01158 |
|  |  | *Selenomonas.1* | -1.476 | <0.00001 |
|  |  | *Veillonellaceae* UCG.001 | -1.213 | 0.03759 |
|  | Proteobacteria | *Ruminobacter* | -1.774 | <0.00001 |
|  | Synergistetes |  | -1.411 | <0.00001 |
|  |  | *Pyramidobacter* | -1.562 | <0.00001 |

^1^ CON, non-supplementation; FAL, 2.25%DM calcium salt of long-chain fatty acid supplementation; FAH, 4.50%DM calcium salt of long-chain fatty acid supplementation; FUM, fumarate supplementation; MON, monensin supplementation. ^2^ Phylum and genus exhibited significant differences (adjusted P < 0.05) identified using DESeq2 with ≥1% relative abundance in more than one treatment. ^3^ The p-value was adjusted using the Benjamini-Hochberg procedure.
